# Synthesis, Experimental and Computational Evaluation of SERAAK1 as a 5-HT_2A_ Receptor Ligand

**DOI:** 10.3390/molecules30102165

**Published:** 2025-05-14

**Authors:** Agata Zięba, Ewa Kędzierska, Michał K. Jastrzębski, Tadeusz Karcz, Agnieszka Olejarz-Maciej, Agata Sumara, Tuomo Laitinen, Tomasz M. Wróbel, Emilia Fornal, Marián Castro, Agnieszka A. Kaczor

**Affiliations:** 1 Department of Synthesis and Chemical Technology of Pharmaceutical Substances with Computer Modeling Laboratory, Faculty of Pharmacy, Medical University of Lublin, 4A Chodźki St., 20093 Lublin, Poland; michal.jastrz1998@gmail.com (M.K.J.); tomasz.wrobel@umlub.pl (T.M.W.); 2 Department of Pharmacology and Pharmacodynamics, Faculty of Pharmacy, Medical University of Lublin, 4A Chodźki St., 20093 Lublin, Poland; 3 Department of Technology and Biotechnology of Drugs, Faculty of Pharmacy, Jagiellonian University, Medical College, Medyczna 9, 30688 Kraków, Poland; t.karcz@uj.edu.pl (T.K.); agnieszka.olejarz@uj.edu.pl (A.O.-M.); 4 Department of Bioanalytics, Medical University of Lublin, Jaczewskiego 8b St., 20-090 Lublin, Poland; agata.sumara@umlub.pl (A.S.); emilia.fornal@umlub.pl (E.F.); 5 School of Pharmacy, University of Eastern Finland, Yliopistonranta 1, P.O. Box 1627, 70211 Kuopio, Finland; tuomo.laitinen@uef.fi; 6 Department of Pharmacology, Center for Research in Molecular Medicine and Chronic Diseases (CIMUS), Universidade de Santiago de Compostela, Avda de Barcelona, 15782 Santiago de Compostela, Spain; marian.castro@usc.es; 7 Instituto de Investigación Sanitaria de Santiago de Compostela (IDIS), Travesía da Choupana s/n, 15706 Santiago de Compostela, Spain

**Keywords:** 5-HT_2A_ receptor, ADMET, antidepressants, antipsychotics, in vivo studies, molecular modeling

## Abstract

Many drug discovery efforts have identified potentially promising molecules; however, a common limitation of these reports is the lack of further experimental confirmation of pharmacokinetic properties and behavioral effects of discovered compounds. In this study, we aim to address this limitation. Therefore, we build on our previous virtual screening campaign by synthesizing, analyzing in silico, and evaluating experimentally the SERAAK1 compound, which was initially identified as a ligand for 5-HT_1A_, 5-HT_2A_, and D_2_ receptors. Through these investigations, we discovered that SERAAK1 binds to the orthosteric pocket of the 5-HT_2A_ receptor in a similar mechanism to that known for marketed antipsychotic medications. Molecular dynamics simulations revealed that the SERAAK1 compound remains stable in the orthosteric binding pocket of the 5-HT_2A_ receptor. The determination of the ADMET parameters indicated the directions for further optimization of the compounds. In vivo studies demonstrated the anxiolytic and antidepressant properties of the SERAAK1 compound.

## 1. Introduction

The serotonin system (5-HT system) plays a crucial role in regulating a wide range of physiological functions such as mood, cognition, and appetite [1]. Its involvement in these processes has made it a central focus of scientific research for decades. Interestingly, certain components of the 5-HT system, e.g., 5-hydroxytryptamine 2A (5-HT_2A_) receptors, have emerged as key molecular targets, validated for the treatment of challenging and debilitating conditions like schizophrenia, depression, anxiety, and neurodegenerative disorders [2,3,4].

The 5-HT_2A_ receptor is predominantly a cortex receptor [5,6] and is involved in the regulation of several physiological and pathological states, such as impulsivity and aggression [7], anxiety and stress [8], learning and cognition [9], as well as mood and depression [6]. The 5-HT_2A_ receptor antagonism is one of the key mechanisms of action of antipsychotics, in particular atypical antipsychotics, such as clozapine, olanzapine, and quetiapine. This 5-HT_2A_ receptor blockade can help to reduce symptoms of schizophrenia and may also enhance the effects of antidepressants in treating depression and anxiety. Antipsychotics that display the 5-HT_2A_ receptor antagonism can relieve the negative symptoms of schizophrenia (e.g., anhedonia), diminish extrapyramidal syndrome (EPS), and decrease hyperprolactinemia [10]. On the other hand, the atypical antidepressant mirtazapine is a 5-HT_2A_ receptor antagonist and is effective in treating major depressive disorder. Some studies suggest that 5-HT_2A_ receptor antagonists can augment the effects of SSRIs, particularly in treatment-resistant depression [11]. Finally, psychedelics, like psilocybin and LSD, are 5-HT_2A_ receptor agonists that have shown promise in treating depression and anxiety in clinical trials [12,13]. The 5-HT_2A_ receptor is also significant in regulating learning and memory [14,15]. Following this, polymorphisms of the human *HTR2A* gene are associated with altered memory processes [14]. Furthermore, the regional distribution of 5-HT_2A_ receptors can be predictive of the memory capacities that are sensitive to serotonin manipulation [14]. Many marketed drugs affect cognitive functions by the 5-HT_2A_ receptor-mediated mechanism. Ritanserin, a 5-HT_2A_ receptor antagonist (used as an anxiolytic, antidepressant, antiparkinsonian agent, and antihypertensive agent), improves memory acquisition in conditioned olfactory training [16]. Ketanserin, another 5-HT_2A_ receptor antagonist used mainly as an antihypertensive drug, improves memory consolidation in autoshaping learning tasks [15] while deteriorating memory acquisition in passive avoidance paradigms [17]. Interestingly, in several behavioral tests, LSD, a 5-HT_2A_ receptor agonist, improved memory acquisition [18,19].

Despite the availability of medications targeting the serotonin systems, many of the available treatments are associated with the development of severe side effects or report limited efficacy [20]. This limitation has fueled the ongoing efforts aimed at developing alternative therapies that can more effectively diminish the symptoms of these disorders. Among a wide array of techniques applied in this process, rational drug discovery is of particular interest. These strategies take advantage of available computational tools to identify potential drug candidates. However, a major bottleneck of this process remains in the accurate prediction of both efficacy and safety profiles of virtually delivered hits. In practice, computational studies must be followed by extensive experimental work to precisely characterize the properties of the compounds [21].

**Figure 1:**
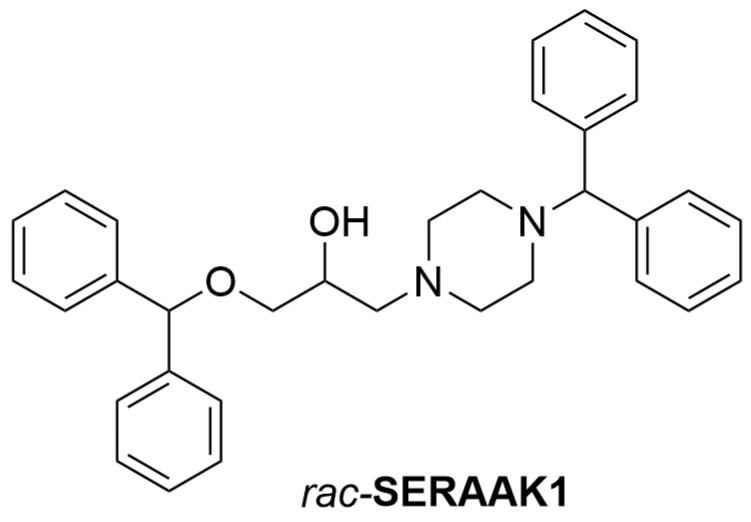
Compound SERAAK1.

Here, we report a continuation of a structure-based virtual screening campaign aimed at discovering novel 5-HT_2A_ receptor ligands. In the previous study, we identified promising virtual hits through docking-based virtual screening, initially selecting 6 compounds based on their affinity and efficacy values determined in in vitro assays [22]. Of particular attention was compound (1-(benzhydryloxy)-3-(4-benzhydrylpiperazin-1-yl)propan-2-ol), here referred to as SERAAK1 (Figure 1). Radioligand binding assays confirmed its affinity at 5-HT_2A_ (*K_i_* 826 ± 51 nM), 5-HT_1A_ (*K_i_* 1425 ± 108 nM), and D_2_ (*K_i_* 1291 ± 323 nM) receptors, while further functional studies found it to be an antagonist of the 5-HT_2A_ receptor (*K_b_* 462 nM) [22].

Building on these findings, we now present a comprehensive description of the properties of SERAAK1. Specifically, our approach integrates chemical synthesis, ADME profiling, in vivo studies, and molecular dynamics (MD) simulations, providing more thorough characterization of the properties of the abovementioned molecule. Ultimately, our goal is to bridge the gap between computational predictions and experimental validation, delivering crucial insights into the therapeutic potential of this molecule, possibly guiding further optimization campaigns.

## 2. Results

### 2.1. Computational Studies on the Compound SERAAK1

Our previous studies have shown that SERAAK1 exerts its effects through the antagonism of the 5-HT_2A_ receptor [22]. Accordingly, this part of the computational investigation focused mainly on deciphering its precise mechanism of action at the 5-HT_2A_ receptor.

#### 2.1.1. Molecular Docking to the Structure of the 5-HT_2A_ Receptor in an Inactive Conformation

**Figure 2:**
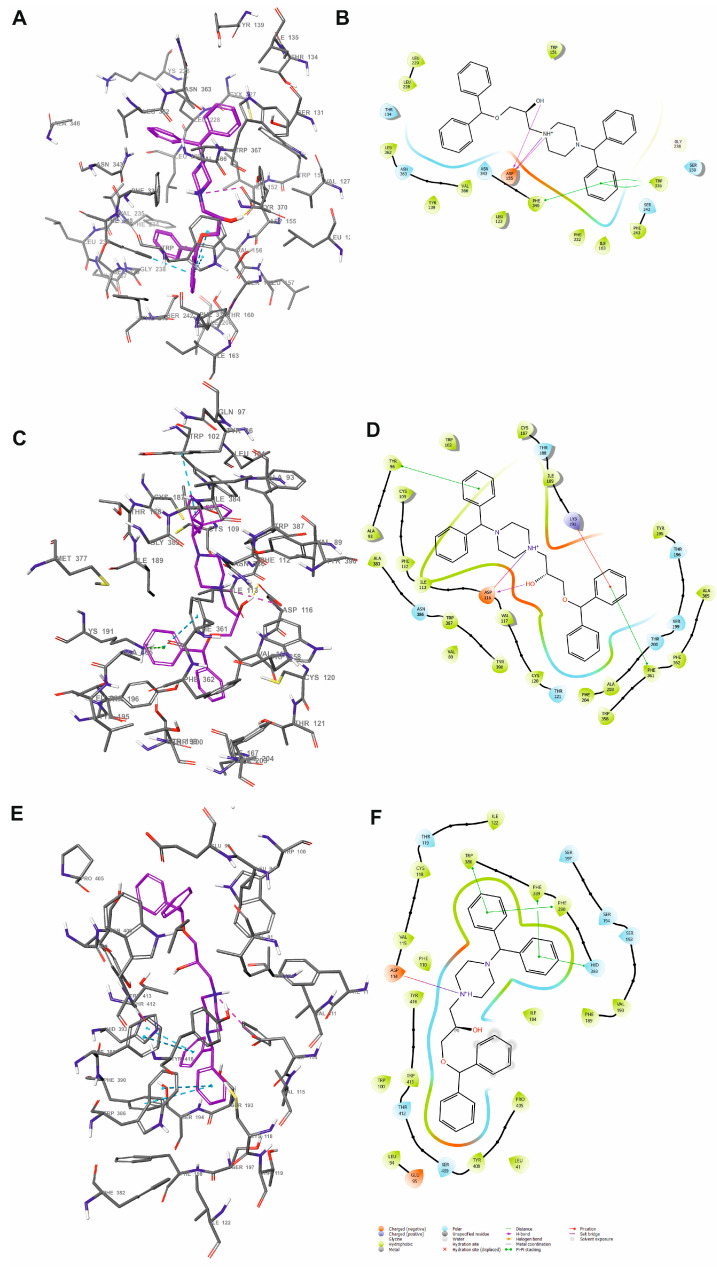
SERAAK1 docked to the orthosteric binding pocket of the 5-HT_2A_ (**A**,**B**), 5-HT_1A_ (**C**,**D**) and D_2_ receptors (**E**,**F**). (**A**,**C**,**E**) 3D overview of the ligand in the binding pocket. Ligand shown as sticks with magenta carbon atoms. Protein shown in stick representation with gray carbon atoms. Non-polar hydrogen atoms omitted for clarity. Ligand-protein interactions are shown as dashed lines (hydrogen bonds—yellow, salt bridges—magenta, π-π stacking—blue). (**B**,**D**,**F**) 2D overview of the ligand in the binding pocket.

A molecular docking study was performed using an inactive template of the 5-HT_2A_ receptor, specifically PDB template 6A93 [23,24]. This procedure revealed several interactions formed between the structure of the ligand and residues from the binding pocket of the receptor. A schematic representation of the predicted binding pose is presented in Figure 2A,B. As anticipated, the main anchoring point in the analyzed complex was the conserved Asp155 (3.32 in Balesteros–Weinstein numbering), which interacted with the protonatable nitrogen atom of the ligand [25]. Furthermore, the hydrophobic moieties of the ligand were predicted to extend deeply into the bottom cleft of the receptor, interacting with residues such as Trp336^6.48x48^ and Phe340^6.52x52^. The ligand-receptor complex, obtained in this part of the study, was subsequently embedded in a lipid bilayer and forwarded to MD simulations.

To study the selectivity of SERAAK1 towards 5-HT_1A_ and D_2_ receptors, molecular docking was also performed. In the case of the 5-HT_1A_ receptor, the structure in active conformation in complex with aripiprazole (PDB ID 7E2Z) [26] was used, and for the D_2_ receptor, the inactive conformation structure in complex with risperidone (PDB ID: 6CM4) [27] was utilized. The main pattern of interactions with Asp^3.32x32^ as the main anchoring point for the protonatable nitrogen atom of the ligand was identical in all the studied receptors (Figure 2A–F). The pose of SERAAK1 in the 5-HT_1A_ receptor (Figure 2C,D) corresponds to those found for the 5-HT_2A_ receptor (Figure 2A,B), while in the D_2_ receptor the ligand is about 180 degrees rotated (Figure 2E,F). It should be stressed that among the requested 50 poses for the SERAAK1-D_2_ receptor complex, only 17 poses were generated, and only one involved the interaction of its protonatable nitrogen with Asp^3.32x32^. It can explain the significantly lower affinity of SERAAK1 towards D_2_ in comparison to the 5-HT_2A_ receptor. Moreover, in the final SERAAK1-D_2_ receptor complex, there is no hydrogen bond between the hydroxylic group of the ligand and Asp^3.32x3.32^, which is found in both complexes with the serotonin receptors. The receptor affinity was also reflected by the Glide docking score, which was −9.970 for the 5-HT_2A_ receptor, −6.611 for the 5-HT_1A_ receptor and −4.686 for the D_2_ receptor.

#### 2.1.2. Molecular Dynamics Simulation of the SERAAK1 Complex with the 5-HT_2A_ Receptor

**Figure 3:**
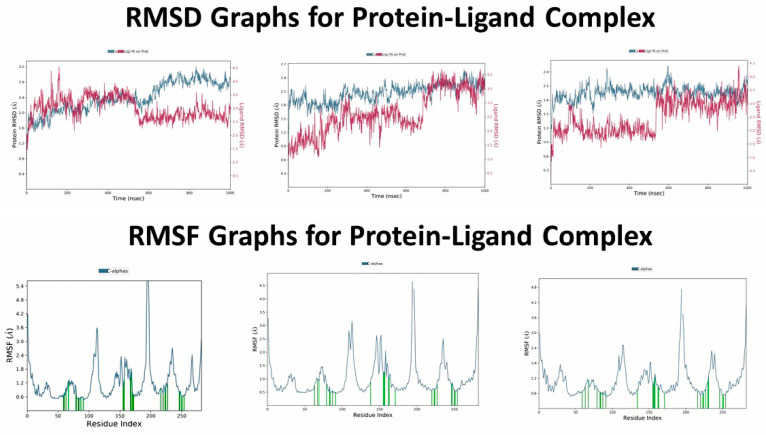
Root mean square deviation (upper figure) and root mean square fluctuation (bottom figure) graphs depicting the outcomes of the molecular dynamics simulation for the complex of the 5-HT_2A_ receptor and SERAAK1. In the upper row, the blue plot depicts the RMSD changes in a protein backbone (left *y*-axis), whereas the pink plot tracks fluctuations in ligand RMSD (right *y*-axis). The bottom row depicts local changes in the flexibility of the protein structure, with peaks indicating regions of increased mobility. Protein residues involved in ligand binding were marked with green vertical bars.

Collected trajectories were further analyzed to gain insight into the binding mechanism of the SERAAK1. Primarily, a root mean square deviation (RMSD) analysis, measuring the extent of displacement in the structure of the protein by comparing the starting conformation to the final position of the complex after the simulation, was performed. Smaller RMSD values are generally preferred, as they indicate greater stability of the simulated complex [28]. In this case, SERAAK1 yielded RMSD values lower than 5 Å, suggesting that the system was well equilibrated and did not undergo any significant conformational changes (Figure 3). Subsequently, a root mean square fluctuation (RMSF) analysis was conducted to assess the flexibility of the protein structure throughout the simulation. It revealed that certain regions, particularly loop regions, displayed more dynamic behavior due to their inherent flexibility. In contrast, the helical regions of the protein remained relatively rigid, resulting in lower RMSF values.

**Figure 4:**
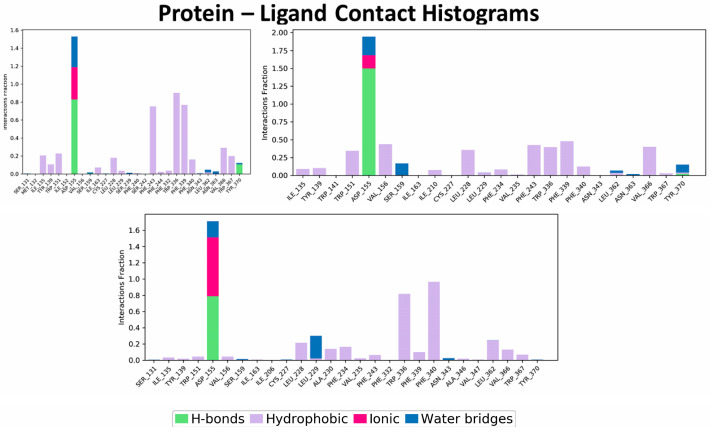
Histograms of ligand–receptor contacts of simulated replicas encompassing complexes of the SERAAK1 and the 5-HT_2A_ receptor. The stacked bar charts are normalized over the course of the trajectory. Values exhibiting 1.0 are possible since some protein residues may participate in multiple contacts of the same subtype with the ligand.

SERAAK1 adopted an extended conformation in the binding pocket of the 5-HT_2A_ receptor. This complex was stabilized by interactions with numerous residues, as indicated in Figure 4. Notably, in the case of all simulations, the interaction with Asp155^3.32x32^ was considered the most persistent one, formed by various interaction types, e.g., hydrogen bonds, water bridges and ionic contacts. These diverse forms of interactions suggest a stable and dynamic engagement between SERAAK1 and this residue and further emphasize its significant role in the modulation of signaling via 5-HT_2A_ receptors. Additionally, this compound formed hydrophobic interactions with toggle-switch Trp336^6.48x48^, as well as Phe339^6.51x51^ and Phe340^6.52x52^. These interactions have been extensively described in the literature in the context of their importance in the activity modulation among 5-HT_2A_ receptor antagonists [24]. More importantly, these residues are conserved among other GPCRs, as in this protein superfamily, similar residues often participate in ligand recognition and receptor activation processes [29]. The implications of these findings extend to the broader understanding of the mechanism of action of SERAAK1, suggesting its possible activity across multiple GPCR subtypes.

As a final step of the process, the principal component analysis (PCA) was performed on trajectories collected from three simulated replicas to gain a deeper understanding of the motion of the complex. This approach enabled the extraction of the most essential components of protein dynamics. The results of this analysis are graphically represented in Figure S1 in the Supplementary Material. Notably, the first three principal components accounted for approximately 35% of the protein backbone movement. Analysis of porcupine plots revealed that these motions were predominantly located in loop regions and adjacent helices, highlighting the crucial role of conformational flexibility in these areas for spatial dynamics.

### 2.2. Synthesis of SERAAK1

**Scheme 1:**
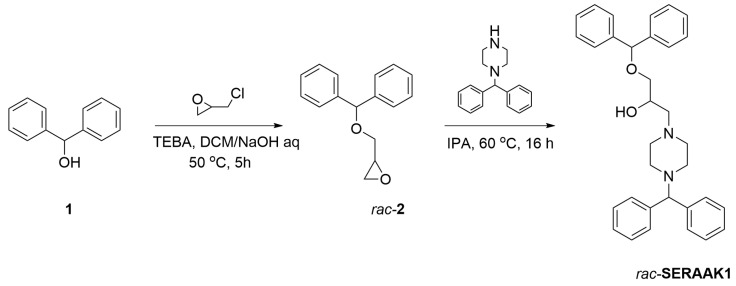
Synthetic route leading to SERAAK1.

To evaluate the pharmacological effects of SERAAK1 in animal models and assess its basic in vitro pharmacokinetic properties, a synthetic route was developed to obtain a pure in-house sample of the compound. SERAAK1 was synthesized via a concise two-step process (Scheme 1). In the first step, diphenylmethanol **1** was reacted with racemic epichlorohydrin in the presence of the phase-transfer catalyst TEBA (Benzyltriethylammonium chloride), yielding epoxide **2**. This intermediate was then subjected to nucleophilic substitution with diphenylmethylpiperazine, affording racemic SERAAK1. The resulting mixture of enantiomers was used in subsequent studies without chiral separation.

### 2.3. ADMET Evaluation of SERAAK1

Understanding ADMET features of a molecule is essential in drug discovery, as these properties determine the bioavailability of the compound and, ultimately, its safety, efficacy, and therapeutic potential [30]. Accordingly, the next phase of the investigation focused on assessing the impact of SERAAK1 on the activity of cytochrome P450 isoforms 3A4 and 2D6, evaluating its cytotoxicity in hepatoma (HepG2) and neuroblastoma (SH-SY5Y) cell models, and investigating its ability to penetrate biological membranes using the PAMPA (Parallel Artificial Membrane Permeability Assay).

#### 2.3.1. Cytochrome P450 Enzyme Activity Assay

Briefly, the effect of SERAAK1 on CYP3A was determined using a luminescent luciferase-based assay. A 10 µM concentration of the compound was tested in two experiments, each with three replicates. The resulting activity was expressed as a percentage of inhibition of the enzyme relative to controls: (i) complete inhibition using the reference inhibitor ketoconazole (IC_50_: 156 nM); (ii) baseline activity with the solvent control (0.2% DMSO). Data obtained in this screening is presented in Table 1.

**Figure 5:**
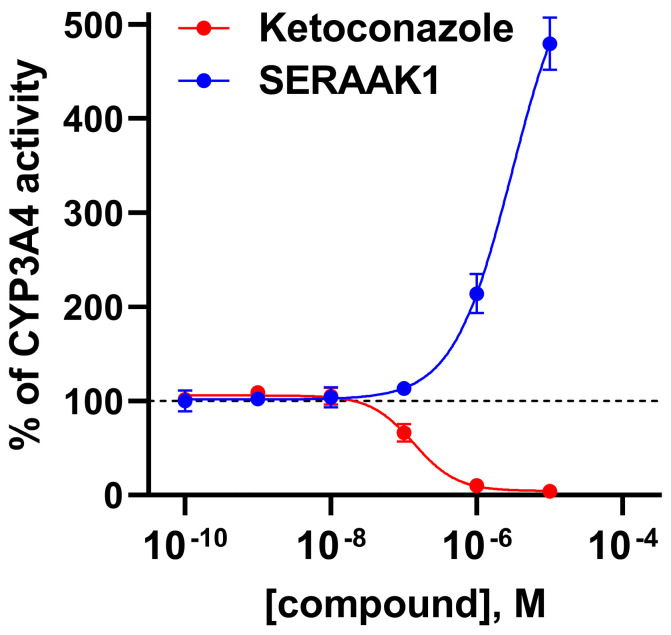
Dose–response curves for induction of the cytochrome P450 3A4 isoform for ketoconazole (reference compound; red) and SERAAK1 (test compound; blue).

SERAAK1 exhibited a strong stimulatory effect on the CYP3A4 complex, leading to the 3,5-fold increase in the enzyme function. Subsequently, this procedure was extended to a wider range of concentrations with the goal of determining the dose–response curve and calculating the EC_50_ value. Unfortunately, due to the fact that in the concentration range used, up to 10 µM, the maximum effect of CYP3A4 stimulation was not achieved, it was not possible to estimate the EC_50_ for this compound. Regardless of the above observation, SERAAK1 at concentrations > 100 nM was found to induce CYP3A4 in a dose-dependent manner (Figure 5).

**Figure 6:**
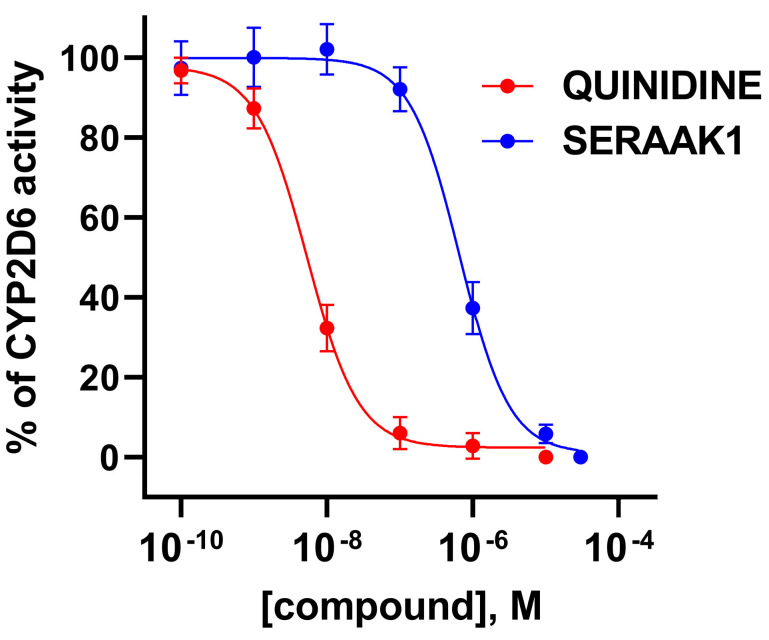
Dose–response curves for inhibition of the cytochrome P450 2D6 isoform for quinidine (reference compound; red) and SERAAK1 (test compound; blue).

The effect of a compound on a CYP2D6 isoform was evaluated using a similar protocol. However, distinct reference samples were used; namely, complete inhibition was achieved with 10 µM of quinidine, whereas baseline activity was observed in 0.2% of DMSO in a phosphate buffer. The outcome of this evaluation is summarized in Table 2. SERAAK1 demonstrated a strong inhibitory activity against CYP2D6, reducing enzyme function by 95% at 10 µM. Further dose–response analysis revealed that SERAAK1 inhibited CYP2D6 in a submicromolar range (Figure 6). Due to the substantial strength of this effect, SERAAK1 was forwarded to an evaluation in a wider range of concentrations. The IC_50_ parameter was determined in this experiment and further compared with the result of a reference drug—quinidine. Interestingly, SERAAK1 inhibited the 2D6 isoform in a submicromolar concentration range, which, in combination with its strong activating effect on the 3A4 isoform, suggests its significant differences in selectivity toward both isoforms. This duality suggests that SERAAK1 may have a substantial impact on drug metabolism, potentially changing the pharmacokinetics of co-administered substances [31].

#### 2.3.2. Cytotoxicity Assays

**Figure 7:**
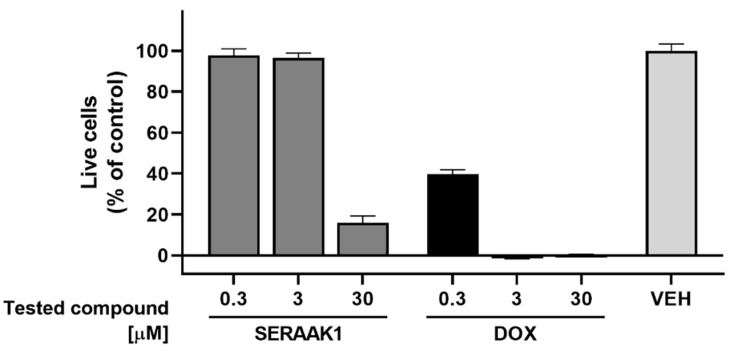
The cytotoxic effects of the tested compounds were evaluated by assessing the percentage of viable cells in the HepG2 cell line. Each assay was conducted twice, with three replicates per experiment. Doxorubicin (DOX) was used as the reference compound for comparison.

**Figure 8:**
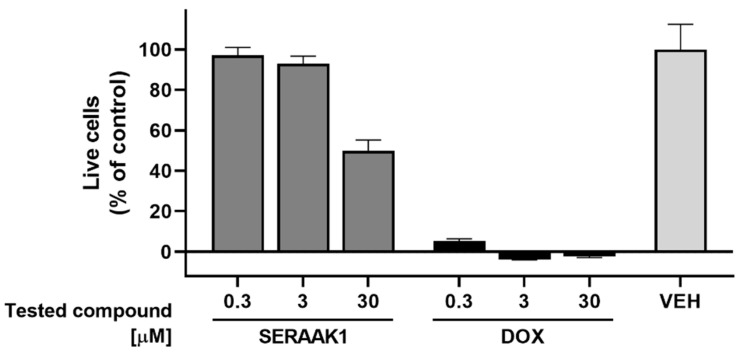
The cytotoxic effects of the tested compounds were evaluated by assessing the percentage of viable cells in the SH-SY5Y cell line. Each assay was conducted twice, with three replicates per experiment. Doxorubicin (DOX) was used as the reference compound for comparison.

The cytotoxic properties of SERAAK1 were tested in two human cell lines, selected based on their relevance to the study objectives—the HepG2 and SH-SY5Y. Figure 7 and Figure 8 depict the results obtained in the cytotoxicity assays performed using the MTS. In the highest concentration tested, SERAAK1 inhibited the proliferation of cells from the SH-SY5Y line (% inh. 50 ± 5), while its cytotoxic effect was significantly higher on HepG2 cells (% inhibition 84 ± 3).

#### 2.3.3. Permeability Assays

The PAMPA assay was utilized to evaluate the ability of SERAAK1 to passively diffuse through a lipid-infused artificial membrane [32]. The results, gathered in Table 3, were expressed as permeability coefficient Pe, calculated according to the formulas described elsewhere [33]. The following reference compounds were used in this study: well-permeable caffeine and poorly permeable sulpiride. Overall, SERAAK1, a piperazine derivative containing two benzhydryl groups, did not permeate passively through a lipid bilayer utilized in this PAMPA test.

### 2.4. Behavioral Studies

A synthesized sample of SERAAK1 was additionally forwarded to behavioral studies aiming to elucidate its therapeutic potential in diminishing symptoms of central nervous system (CNS) disorders. This section of the manuscript delves deeper into the outcomes of these studies and interprets these data in the context of the pharmacological usefulness of SERAAK1.

#### 2.4.1. SERAAK1 Effects on Spontaneous Locomotor Activity in Mice

**Figure 9:**
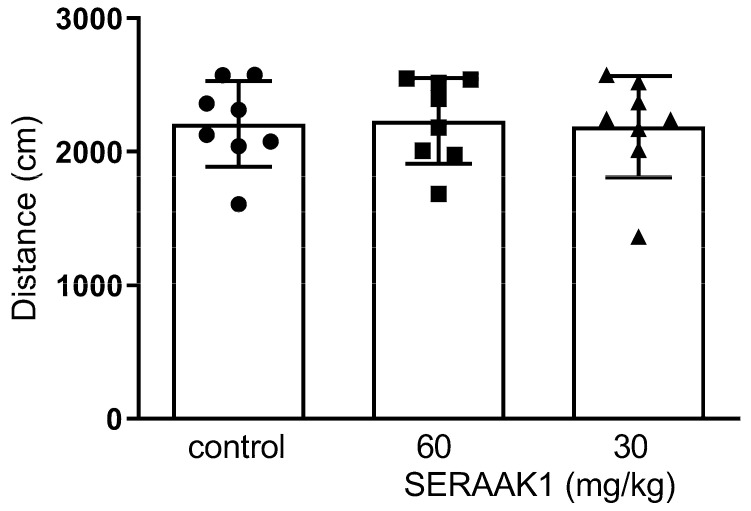
The influence of the tested compound SERAAK1 (60 and 30 mg/kg) on the spontaneous locomotor activity of mice. The investigated compound was injected i.p. 60 min before the test. Locomotor activity was measured for 20 min. Data are expressed as mean ± SEM values of the 1 independent experiment.

Spontaneous locomotor activity is a meaningful parameter helping to understand the physical and mental status of laboratory animals, providing insights into general activity levels, arousal and potential sedative or stimulant effects of administered substances [34]. The effect of SERAAK1 on the spontaneous locomotor activity in mice is presented in Figure 9. We measured the distance traveled by the mice during the 20 min observation period. One-way ANOVA did not show any significant changes in locomotor activity of mice 20 min after administration of the compound SERAAK1 [ANOVA: F(2, 21) = 0.03227]. These results indicate that acute administration of the compound does not alter baseline locomotor activity of laboratory animals, which suggests that the compound itself does not exert a sedative or enhancing effect at the doses tested.

#### 2.4.2. SERAAK1 Effects on Motor Coordination in Mice

**Figure 10:**
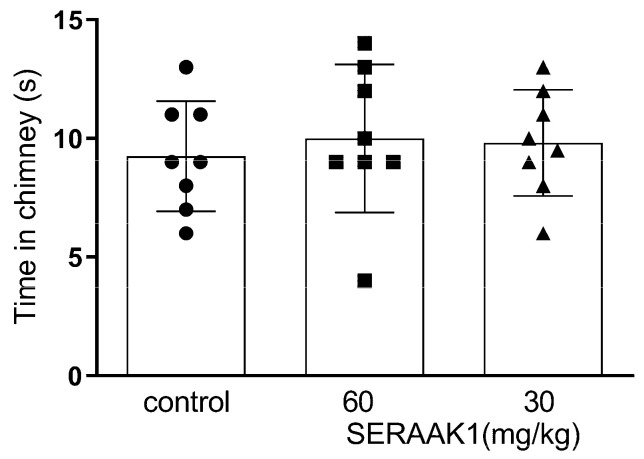
The influence of the tested compound SERAAK1 (60 and 30 mg/kg) on motor coordination in mice was evaluated in a chimney test. Investigated compounds were injected i.p. 60 min before the test. Data are expressed as mean ± SEM values of the 1 independent experiment.

**Figure 11:**
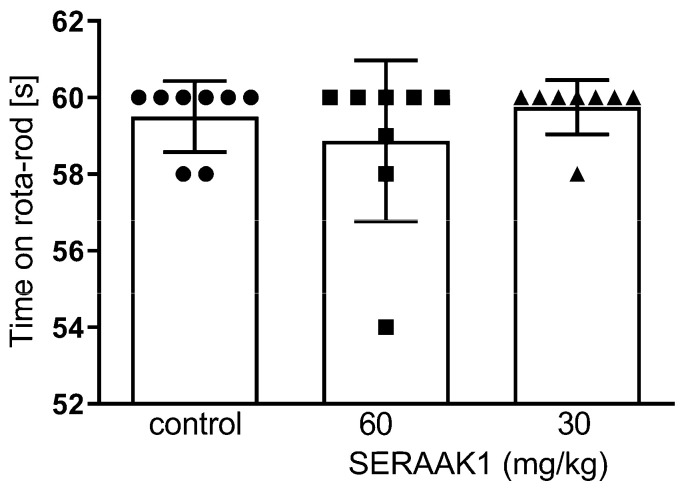
The influence of the tested compound SERAAK1 (60 and 30 mg/kg) on motor coordination in mice was evaluated in a rota-rod test. Investigated compounds were injected i.p. 60 min before the test. Data are expressed as mean ± SEM values of the 1 independent experiment.

The one-way ANOVA did not reveal any statistically significant effects of SERAAK1 on motor coordination in mice at doses of 30 mg/kg and 60 mg/kg. This conclusion was supported by results from two widely used behavioral tests: the chimney test (Figure 10) and the rotarod test (Figure 11), conducted 60 min after the substance administration [35,36]. In the chimney test, we measured the time it took the mice to exit the chimney, and in the second test, the time the animals stayed on the rod rotating at a constant speed of 18 rpm.

#### 2.4.3. Effect of Acute Administration on Anxiety-like Behavior

**Figure 12:**
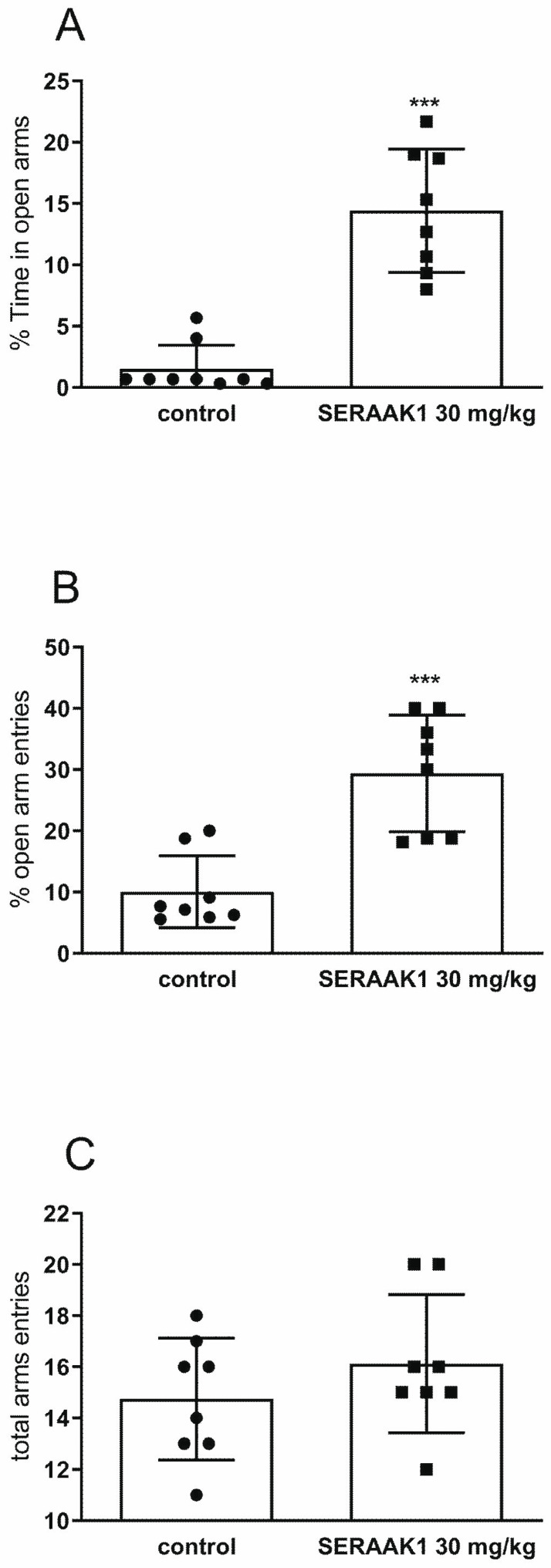
The influence of the investigated compound SERAAK1 (30 mg/kg) on elevated plus maze performance in mice—percentage of time spent in open arms (**A**), the percentage of the open arm entries (**B**), and total arm entries (**C**). The investigated compound was injected i.p. 60 min before the test. The results are expressed as mean ± SEM of the 1 independent experiment. *** *p* < 0.001 vs. control (Student’s *t*-test).

The effect of SERAAK1 on anxiety-like behavior in mice was assessed using the elevated plus maze test (EPM) [37,38]. In this test we measured three parameters: time spent in the open arms of the maze, the number of entries into the open arms—expressed as a percentage of the time spent/entries in both types of maze arms and the total number of entries into the open and closed arms of the maze, which is an additional measure of the animals’ locomotor activity. The anxiolytic activity of mice after SERAAK1 administration was expressed as the percentage of time spent in the open arms of the maze and the number of entries into these arms. Statistical analysis with a *t*-test indicated a significant influence of acute administration of SERAAK1 (30 mg/kg) on anxiety responses. This was evident from the increased percentage of time spent on the open arms (by 12.9%, *p* < 0.001; Figure 12A) and the higher percentage of open arm entries (by 19.34%, *p* < 0.001; Figure 12B) in animals treated with this substance.

These findings suggest that SERAAK1 exhibited an anxiolytic-like effect, as both measures reflected reduced anxiety levels. Interestingly, the tested compound at the dose of 30 mg/kg did not change the overall mobility of animals, as evidenced by the unaltered number of total arm entries (open and closed arms combined). Thus, regardless of the anxiolytic effect, it did not alter the general mobility of treated mice.

#### 2.4.4. Total Immobility Duration in the Forced Swim Test

**Figure 13:**
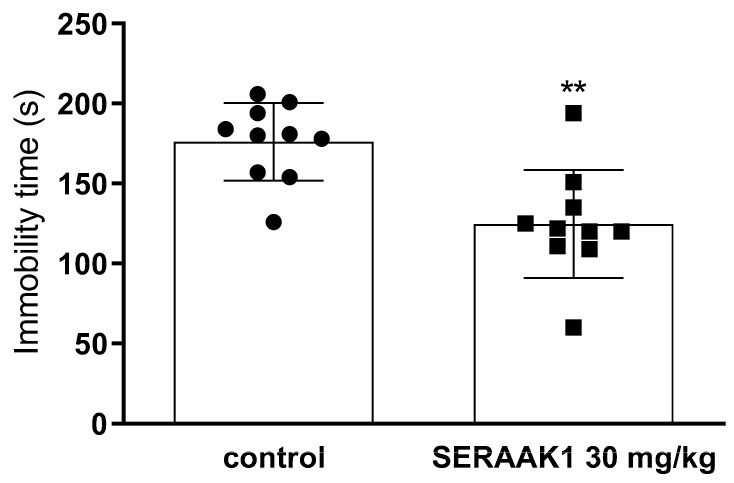
The influence of SERAAK1 (30 mg/kg) on the total duration of immobility in the forced swim test in mice. The investigated compounds were administered i.p. 60 min before the test. The values represent means ± SEM of the 1 independent experiment. ** *p* < 0.01 vs. control (Student’s *t*-test).

The forced swim test (FST) is commonly used in rodent laboratory models to assess the efficacy of a potential antidepressant drug in alleviating or preventing depressive-like states [39]. In the FST, we estimated the immobility time of animals, illustrating it despite mice. In this study, a *t*-test was used to assess the statistically significant effect of acute administration of SERAAK1 (30 mg/kg) on mice behavior in the FST. Results showed a significant reduction in the immobility time in the SERAAK1-treated group compared to the control group (by 29.2%, *p* < 0.01) (Figure 13), which may indicate the antidepressant-like properties exhibited by this compound.

#### 2.4.5. Effect of Acute Administration on Amphetamine-Induced Hyperactivity

**Figure 14:**
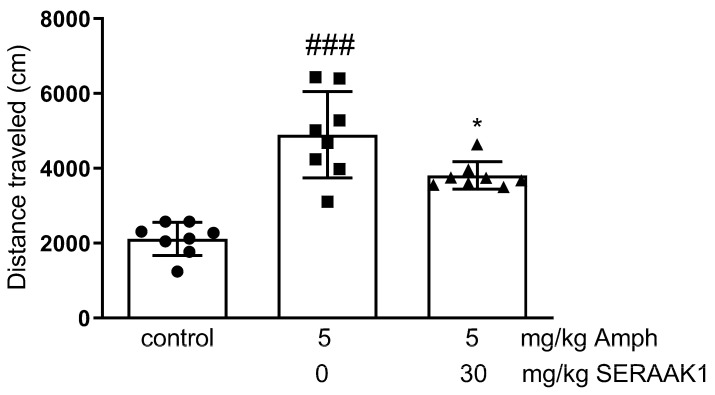
Effects of acute administration of SERAAK1 (30 mg/kg) on amphetamine-induced hyperactivity in mice. Appropriate groups of trial animals received injections of SERAAK1 (30 mg/kg), amphetamine (5 mg/kg), and vehicle i.p. on Day 1 immediately after the test, and the rodents were retested 24 h later (i.e., on Day 2). The results are expressed as mean ± SEM of the 1 independent experiment. * *p* < 0.05 vs. amphetamine-treated group in Dunnett’s test; ### *p* < 0.001 vs. control in Dunnett’s test.

In this test, we measured the distance traveled by the mice during the 20 min observation period. Interestingly, in the case of SERAAK1, statistical analysis demonstrated significant effects on locomotor activity after treatment in combination with amphetamine (two-way ANOVA: pretreatment [F (1, 7) = 80.09; *p* < 0.0001], and the interaction effect between pretreatment and treatment [F (1, 7) = 5665, *p* = 0.0489]). Pretreatment concerns the administration of amphetamine, and treatment concerns the tested compound. The administration of amphetamine increased the locomotor activity of mice, measured as the distance traveled (in cm) vs. the saline-treated group (*p* < 0.001). Moreover, the post hoc Dunnett’s test showed that SERAAK1 (30 mg/kg) co-administered with amphetamine (5 mg/kg) decreases amphetamine-induced hyperactivity when compared to the amphetamine-treated group (by 22.15%, *p* < 0.05) (Figure 14).

## 3. Discussion

Here, we present the results of a campaign focused on investigating the properties of a virtually derived compound, SERAAK1, which has previously been shown to bind to 5-HT_2A_, 5-HT_1A_, and D_2_ receptors. Various experimental and computational techniques were used to gain a more detailed understanding of the potential of the compound and identify possible directions to explore in further optimization campaigns. Here, we focused on the molecular level.

Specifically, MD simulations suggested that the binding mode of SERAAK1 is similar to that of atypical antipsychotics. It adopts an extended conformation in the binding pocket, reaching the bottom hydrophobic cleft of the receptor [24]. Moreover, frequent interactions with GPCR conserved residues suggest that additional receptor studies may be necessary in order to fully decipher the therapeutic potential of this ligand, as well as to identify possible side effects resulting from its multi-target profile.

Subsequently, in vitro ADMET evaluation was performed to gain a deeper understanding of the properties of this compound in human cells. Initially, special emphasis was placed on deciphering its influence on CYP3A4 and CYP2D6 enzymes, which are among the most important cytochrome enzyme isoforms identified in the human body [31,40,41,42]. In particular, CYP3A4 is responsible for the metabolism of approximately 50–60% of marketed drugs, while CYP2D6 is involved in the metabolism of substances such as, e.g., antidepressants, opioids, and beta-blockers [43,44]. Understanding how a new molecule influences these enzymes is essential for two main reasons: it helps to estimate the risk of developing drug–drug interactions when administered with other substances and predicts the likelihood of adverse reactions, especially for the highly polymorphic CYP2D6 enzyme. Interestingly, regulatory agencies, such as the U.S. Food and Drug Administration, recommend studies evaluating the impact of new drugs on major CYP enzymes, making it a key parameter to investigate even in early stages of the development [45]. In the assays performed, SERAAK1 showed a distinct selectivity profile toward these enzymes. Specifically, it induced the activity of the CYP3A4 isoform and inhibited the CYP2D6 isoform. Since the assays were performed using purified recombinant enzyme preparations, the observed effects reflect direct interactions of SERAAK1 with the catalytic activity of cytochrome P450 isoforms. The inhibition of CYP2D6 activity by SERAAK1 may therefore result from competitive or non-competitive binding at the enzyme’s active site, potentially blocking substrate access or altering the enzyme’s conformation. In contrast, the enhancement of CYP3A4 activity suggests that SERAAK1 may act as an allosteric activator, stabilizing a more catalytically efficient enzyme conformation or facilitating substrate binding and turnover. Such allosteric modulation of CYP3A4 is well-documented in the literature, as this isoform is known for its multiple binding sites and capacity for cooperative binding behavior [46]. Whether SERAAK1 itself is metabolized by CYP enzymes remains an open question for future investigation.

Subsequently, the influence of SERAAK1 on cell viability was evaluated using two specific cell lines: HepG2 and SH-SY5Y. Both models are human-derived, offering more clinically relevant toxicity predictions than non-human models. HepG2 cells, derived from human hepatocellular carcinoma, are a common choice when studying the hepatotoxicity of a molecule [47]. Since the liver is a primary site of drug metabolism and detoxification, evaluating this property can help in estimating the hepatotoxicity of a compound. The latter cell line—SH-SY5Y—comprises neuroblastoma-derived cells that mimic neuronal characteristics [48]. Thus, the use of such a model to study the effect of a compound could be beneficial in understanding a potential neurotoxicity of a molecule. The assays performed indicated that SERAAK1 has no toxic influence on these cell lines in lower concentrations (0.3 µM, 3 µM). However, when the dose increased to 30 µM, the effect of the compound was changed. In SH-SY5Y cells, SERAAK1 slightly inhibited cell proliferation, with a mean inhibition rate of 50 ± 5%. In contrast, its cytotoxic effect was significantly more pronounced in HepG2 cells at the same concentration, with an inhibition rate of 84 ± 3%.

Interestingly, the hepatotoxicity of antidepressants and antipsychotics is a topic of ongoing debate. Many of the recently marketed agents are known to cause idiosyncratic, unpredictable and reversible liver injury. Symptoms of the injury may emerge as early as a few days after the first dose or may take up to 6 months to manifest. In many instances, discontinuation of treatment reverses the pathological changes [49]. Regardless of the effects caused by the marketed medications, the potential hepatotoxicity of the compound SERAAK1 raises serious concerns about the safety of the molecule and comprises a feature to address in further optimization campaigns.

Finally, passive permeability of the SERAAK1 was assessed in PAMPA. It has shown an extremely low passive permeability, falling below the measurable threshold for the PAMPA. This finding suggests that SERAAK1 may not be able to efficiently migrate through membranes using passive diffusion, which could limit its bioavailability at the target site and exacerbate side effects related to accumulation in unintended peripheral tissues. However, to fully assess the potential of a compound to migrate through biological membranes, additional assays evaluating other forms of transport could potentially benefit the study.

This compound was also tested in vivo, as our primary goal was to assess the properties of SERAAK1 valuable for the treatment of mood and psychiatric disorders. First, part of these experiments included locomotor activity tests and motor coordination tests—acknowledged as fundamental in central activity investigations of new agents [50]. In these studies, SERAAK1 was administered at doses of 30 and 60 mg/kg, and it did not change the mobility of the animals after 20 min of observation. The same doses were used for tests assessing animal coordination: the rotarod and the chimney test [35,36]. Similarly, SERAAK1 did not impair the behavior of mice on a rotating rod or increase the time to exit the chimney compared to the control group (Figure 10 and Figure 11), suggesting no effects of this compound on muscle tone and no neurotoxic effect.

Anxiety and stress-related disorders are among the conditions that impair performance in daily life and have a high public health cost. Charles Darwin’s preliminary observation that animals and humans have similar traits in expressing emotions opens the possibility of studying the mechanisms of mental disorders in other mammals (mainly rodents). Therefore, the anxiolytic properties of SERAAK1 were studied in the elevated plus maze test (EPM). This test is based on the tendency of mice to stay in sheltered, enclosed, dark spaces and the fear of open spaces. Observations describing a reduced percentage of time spent in the closed arms of the maze may reflect reduced levels of anxiety in mice. The EPM test is considered a precise method for studying changes in anxiety behavior in the context of evaluating the therapeutic properties of new drugs [51]. Interestingly, buspirone—an arylpiperazine compound—shows activity in this test. It has been confirmed that buspirone is a partial agonist of the 5-HT_1A_ receptors and affects serotonin transmission in the limbic structures of the brain, making it effective in generalized anxiety disorder and less effective in reducing panic [52,53]. In conclusion, the results obtained in this part of the in vivo study showed that the compound SERAAK1 was active in the EPM test, significantly increasing the time spent in open arms and increasing the number of open-arm entries. In addition, it could be hypothesized that its anxiolytic effect, as in the case of buspirone, could be due to its interaction with the 5-HT_1A_ receptors because of its high receptor affinity.

The anxiolytic effect of SERAAK1 can also result from its 5-HT_2A_ receptor affinity/antagonism. The study conducted by Motta et al. examined the effects of ketanserin in EPM in rats. It was found that the low dose of ketanserin (0.5 mg/kg) decreased aversion to the open arm of the apparatus, suggesting anxiolytic-like activity of the compound; however, a higher dose of 1 mg/kg resulted in decreased locomotor activity, indicating a potential sedative effect. These results showed that the effect of ketanserin on anxiety-related behaviors is dose-dependent and may involve its antagonistic effect on 5-HT_2A_ receptors [54]. In another study, ketanserin increased open arm exploration in diestrous female rats, indicating an anxiolytic-like effect [55]. Moreover, ritanserin—a relatively selective 5-HT_2A/2C_ antagonist—was found active in animal models of anxiety using natural aversive stimuli and in animal models of depression [56]. A clinical study assessing the efficacy and tolerability of ritanserin compared to lorazepam and placebo in 83 patients diagnosed with generalized anxiety disorder (GAD) was also reported. As a result of the observations, it was determined that patients receiving 10 mg of ritanserin or 4 mg of lorazepam showed significant improvement in anxiety symptoms compared to the placebo group (*p* < 0.05), and interestingly, patients receiving lorazepam reported more frequent sedation and dizziness compared to patients receiving ritanserin, suggesting that it may be a viable alternative to traditional benzodiazepines in the treatment of anxiety disorders [57]. Subsequent studies indicate that modulation of 5-HT_2A_ receptors may influence anxiety-related behaviors in animal models. Specific results are inconsistent and depend on factors such as dosage, receptor selectivity, and the presence of other pharmacological agents. Therefore, the observed anxiolytic activity of SERAAK1 may also result from interactions with 5-HT_1A_ or 5-HT_2A_ receptors [58,59].

The second part of the behavioral studies focused on the evaluation of the antidepressant properties of SERAAK1. For this purpose, the forced swim test (FST) was applied, which is a widely established technique used in experimental pharmacology [60]. It is designed to mimic symptoms of human depression. Selective serotonin reuptake inhibitors are an example of antidepressants active in this test. They block the serotonin transporter (SERT) protein that transports serotonin and reduce its reuptake from the synaptic cleft. Because of their efficacy, they are one of the most commonly used drugs for depression. After administration of SERAAK1 at a dose of 30 mg/kg, mice remained immobile for a shorter period compared to the control group, indicating the antidepressant-like activity of the compound. This observation can result from the affinity/antagonism of the SERAAK1 compound to the 5-HT_2A_ receptor. Similar antidepressive activity is known for atypical antipsychotics, such as olanzapine, or atypical antidepressants, such as mirtazapine, and is attributed to their 5-HT_2A_ receptor antagonism. This effect was also found in an FST for a number of novel compounds exhibiting the 5-HT_2A_ receptor antagonism [61].

Finally, we tested the effect of SERAAK1 on the amphetamine-induced hyperactivity in mice. Amphetamine, a psychostimulant, increases locomotor activity by enhancing neurotransmitter release. This phenomenon is often used as a model to study the effects of novel drugs on motor behavior, dopaminergic signaling, or psychosis-like behaviors in animals [62,63]. Some antipsychotic medications can reverse amphetamine-induced hyperactivity, presumably via interactions with D_2_ and noradrenaline receptors [64]. However, it was also reported that 5-HT_2A_ receptor antagonism reduces hyperactivity induced by amphetamine, cocaine, and MK-801 [65], which is a case of the SERAAK1 compound.

## 4. Materials and Methods

### 4.1. Molecular Modeling

The 3D conformation of the compound SERAAK1 was modeled using the LigPrep module of Schrödinger’s suite of software v. 2021-4, as reported previously [22]. For in silico studies, we used the R enantiomer of the compound, as it was the hit found in the previously reported virtual screening campaign [22]. To screen the Enamine database, we used all the possible combinations of stereoisomers, and we found the R enantiomer of SERAAK1 to be highly scored and found among the top-ranked compounds. To sample ligand protonation states at physiological pH, the Epik module of Schrödinger’s suite of software v. 2021-4 was utilized [66].

Subsequently, the X-ray structure of the serotonin 5-HT_2A_ receptor representing the inactive conformation complexed with atypical antipsychotic risperidone (PDB ID: 6A93) was obtained from RCSB PDB [23,24,67]. To study SERAAK1 selectivity towards 5-HT_1A_ and D_2_ receptors at the molecular level, we also used the cryo-EM serotonin 5-HT_1A_ receptor in complex with aripiprazole (PDB ID: 7E2Z) [26] and the X-ray dopamine D_2_ receptor structure in complex with risperidone (PDB ID: 6CM4) [27]. The protein template was preprocessed using a Protein Preparation Wizard module of Maestro from the Schrödinger suite of software v. 2021-4 [68]. Missing extracellular loops were completed with Yasara Structure as previously reported [69]. Protein representation and co-crystallized ligand structure were utilized for the docking grid generation. The created grid box was centered on the ligand structure, and the most important hydroxyl groups of the protein residues were selected as rotatable.

Molecular docking was performed using Standard Precision (SP) mode with Glide of Schrödinger’s suite of software v. 2021-4, and multiple poses were generated for this receptor-ligand pair [70]. Subsequent filtering aimed to ensure that the final selected pose represents a favorable conformation of the ligand within the binding pocket and depicts a crucial interaction between Asp 3.32 of the receptor and a protonatable nitrogen atom of the ligand.

MD simulations with Desmond were performed for the selected complex. Briefly, it was embedded in a lipid bilayer composed of POPC (1-palmitoyl-2-oleoyl-sn-glycero-3-phosphocholine), hydrated with SPC (simple point charge) waters, and neutralized and enriched by ions to a concentration of 0.15 M NaCl. Protein-ligand systems were subsequently subjected to minimization and relaxation protocols to remove steric clashes and perform system equilibration. MD simulations were carried out up to 1 microsecond per replica, while a total of 3 replicas were generated to enhance the sampling of the conformational space. The output files were preprocessed, trimmed, aligned and exported in “.xtc” format. The Simulation Interaction Diagram tool, part of the Desmond package, (Schrödinger Release 2021-4) was utilized to examine trajectories. For more thorough analysis, including the exploration of dominant motion components, principal component analysis (PCA) was conducted using the trj_essential_dynamics.py script, available in the Schrödinger software (v.2021-4) [71].

### 4.2. SERAAK1 Synthetic Procedure

A round-bottom flask was charged with benzhydrol 1 (25 mmol, 1.0 eq, 4.6 g), triethylbenzylammonium chloride (TEBA) (25 mmol, 1.0 eq, 5.7 g), and epichlorohydrin (75 mmol, 3.0 eq, 7.0 g). The reagents were dissolved in a biphasic 1:1 mixture of concentrated NaOH solution (13 mL, 45%) and DCM (13 mL). The mixture was stirred vigorously to ensure good phase contact under reflux (set to 55 °C). After overnight reaction, a TLC analysis showed completion of the reaction [TLC in a system of 10 and 20% EtOAc]. The reaction was quenched with a large amount of water (1 L). The inorganic precipitate was dissolved afterward. Extraction was performed using DCM and CHCl_3_. The crude product was distilled under reduced pressure (120–125 °C, pump set to 6 mbar), obtaining clear oil (2.3 g, 39%). The key intermediate 2 was directly used for the subsequent step. Under an inert atmosphere (argon), a flask was charged with 2 (1.05 eq, 5.33 mmol, 1.28 g), diphenylmethylpiperazine (1.0 eq, 5.07 mmol, 1.28 g), and 50 mL of isopropanol. The reaction mixture was heated in an oil bath (set to 60 °C) overnight. The compound was converted to hydrochloride by adding concentrated HCl until an acidic environment was observed (pH < 3). The product was triturated with Et_2_O (10 mL) using an ultrasonic bath and filtered. It was purified by column chromatography, eluting first with DCM to separate nonpolar impurities and subsequently with 5% 7N NH_3_/MeOH in DCM, yielding a white solid (240 mg, 10%). Complete spectral (HNMR, CNMR and HRMS) and chromatographic characterization confirmed the identity and purity of the desired compound (Figures S2–S5 in the Supplementary Information).

^1^H NMR (600 MHz, MeOD) δ 7.32 (d, *J* = 7.2 Hz, 4H), 7.26–7.22 (m, 4H), 7.17 (dt, *J* = 17.5, 7.6 Hz, 8H), 7.11 (t, *J* = 7.3 Hz, 4H), 7.06 (t, *J* = 7.4 Hz, 4H), 5.31 (s, 1H), 4.11 (s, 1H), 3.86 (tt, *J* = 18.7, 9.4 Hz, 1H), 3.36–3.27 (m, 2H), 2.60–2.15 (m, 10H).

^13^C NMR (151 MHz, CDCl_3_) δ 142.6 (2C), 142.0 (2C), 128.6 (4C), 128.4 (4C), 127.9 (2C), 127.5 (4C), 127.1 (2C), 127.0 (4C), 84.2 (1C), 76.1 (1C), 71.5 (1C), 66.0 (1C), 61.0 (1C), 53.6 (2C), 51.5 (2C).

### 4.3. ADMET Parameters

#### 4.3.1. Influence on Cytochrome P450 3A4 and 2D6 Activity

Luminescence-based CYP3A4 P450 Glo and CYP2D6 P450 Glo kits (Promega, Madison, WI, USA) were used to assess the impact of SERAAK1 on the activity of two distinct cytochrome P450 isoforms. These assays were performed according to the protocol of the manufacturer and in alignment with the previously described methods [72]. Each assay was performed twice in triplicate. Luminescence signals were measured using a Spark microplate reader (Tecan, Männedorf, Zürich, Switzerland). Obtained results were further evaluated with reference to ketoconazole, a well-studied inhibitor of the CYP3A4 isoform, and quinidine, which acted as a reference compound for CYP2D6.

#### 4.3.2. In Vitro Cytotoxicity Evaluation

The cytotoxic effect of SERAAK1 was evaluated using the colorimetric MTS assay. The study was conducted on HepG2 and SH-SY5Y cell lines, following the same protocol. Cell lines were obtained from the American Type Culture Collection (Manassas, VA, USA) and maintained under standard conditions (5% CO_2_, 37 °C) in DMEM/F12 medium (#31330-038, ThermoFisher Scientific, Waltham, MA, USA), supplemented with 10% FBS (#A5256801, ThermoFisher Scientific) and Penicillin-Streptomycin (#15140-122, ThermoFisher Scientific) until used in the experiment. Cells at passage numbers between 15 and 25 were seeded in 96-well plates at the concentration of 5000 cells/well in 200 μL of medium and incubated for 24 h at 37 °C with 5% CO_2_. The medium was then replaced with fresh medium (120 μL), and serial dilutions of the test compound SERAAK1 (prepared in DMSO and culture medium) were added (80 μL), resulting in final concentrations of 30 μM, 3 μM, and 0.3 μM. Doxorubicin, a known cytotoxic agent, served as the reference compound. In the end, control wells contained cells, medium, and 1% DMSO. Compounds were incubated with cells for 72 h. After incubation, MTS/PBS reagent (5:1 ratio) was added (120 μL/well) following medium removal. Subsequently, plates were incubated at 37 °C, and absorbance was measured using a Spark microplate reader after 1 h for HepG2 and 3 h for SH-SY5Y. Final cell viability was calculated relative to control wells (100% viability) and wells with 10 μM doxorubicin (0% viability). These assays were performed in duplicate.

#### 4.3.3. Membrane Permeability Evaluation

The pre-coated PAMPA Plate System Gentest from Corning (Tewksbury, MA, USA) was used in experiments using the instructions of the manufacturer. SERAAK1 and reference compounds were diluted in the PBS buffer (pH 7.4) to the final concentration of 100 μM and placed on the 96-well donor microplate. The exact quantity of molecules that successfully penetrated from donor to acceptor wells through the phospholipid membrane was estimated after 5 h of incubation using UPLC-MS spectrometry (Waters ACQUITY™ TQD system with the TQ Detector, Waters, Milford, CT, USA). The final permeability coefficient (P_e_, cm/s) calculation was performed using the formula provided by the vendor of the PAMPA Plate System [33].

### 4.4. Behavioral Studies Protocols

The ARRIVE checklist was included during manuscript preparation.

#### 4.4.1. Drugs

The investigated compound (SERAAK1) in all tests was administered intraperitoneally (i.p.), spread in a drop of Tween 80, diluted by an aqueous solution of 0.5% methylcellulose (tylose) and injected 60 min before the tests in a volume of 10 mL/kg. Initial doses of the compound were selected based on available literature and our earlier studies [60,73]. Control groups received tylose injections of the same volume and via the same route of administration.

#### 4.4.2. Animals

All experiments were conducted on a 6-week-old naïve male Swiss mice weighing 24–30 g in the Experimental Medicine Center, Medical University of Lublin, Poland. The animals were housed (four/five per cage) under standard laboratory conditions (room temperature of 22 ± 1 °C and a relative humidity of 50–60% during a 12/12 h light-dark cycle, lights on at 8:00). The animals had free access to laboratory food—pellet diet (LSM, Agropol Motycz, Marynin, Poland) and tap water—except for the short time when they were removed from the cages for testing. All experiments were performed in strict accordance with the EU Directive 2010/63/EU for animal experiments and with the approval of the Local Ethics Committee for Animal Experimentation in Lublin (license number 55/2022, issued on 4 April 2022). Mice were randomized for groups and treatments (the number of mice in each group, n = 8–10).

#### 4.4.3. Spontaneous Locomotor Activity and Amphetamine-Induced Hyperactivity

The automatic device—actimeter Opto-Varimex-4 Auto-Track (Columbus Instruments, Columbus, OH, USA)—was used to measure the locomotor activity of experimental animals. The mice were individually placed in cages for 25 min: the first 5 min for acclimatization and then the next 20 min for observation while the distance (in centimeters) traveled during this time was measured. After each mouse, the cages were cleaned with 10% ethanol.

For the spontaneous locomotor activity study, tylose was administered to the control group and the tested compound. For the amphetamine-induced hyperactivity tests, tylose and the tested compounds and after 30 min, amphetamine (3 mg/kg) was administered. While the test was being performed, animals were individually caged, initially for 5 min of adaptation, followed by 20 min of actual mobility testing.

#### 4.4.4. Motor Coordination

The effects of SERAAK1 were also measured in the rotarod and chimney tests [35,36]. In the first test, the ability of mice to balance on a rotating rod (18 rpm, constantly) was assessed for 1 min. The second test was carried out using a polymer tube (named chimney) with an internal diameter of 3 cm and a length of 25 cm. The chimney was placed in a horizontal position, and the animal was led inside. Once the mouse was at the other end, the tube was placed vertically, forcing the rodent to get out only by climbing backwards on the rough surface inside the tube. Prior to the study, animals had been trained for 3 days, and only those that had been able to stay on the rotating rod or leave the chimney within 60 s were included.

#### 4.4.5. Elevated Plus Maze Test (EPM Test)

Anxiety behaviors were measured using the EPM test according to the Lister method [38]. The device was made up of four black-painted, crossed arms arranged in a plus with a central platform (5 × 5 cm). Two arms, 30 × 5 cm, were open, while the other two, 30 × 5 × 15 cm, were closed. Individual types of arms were placed opposite each other. The whole structure was elevated to a height of 38.5 cm above the floor and lit with weak matte red light. The experiment was performed in a quiet, dark room: the mice were individually placed at the central square of the maze, facing the open arm, and their behavior was observed for 5 min by an observer equipped with a stopwatch, unaware of the treatment schedule. The time spent in the EPM open arms, the number of entries into the open arms, and the total number of entries into both types of arms were measured.

#### 4.4.6. Forced Swim Test (FST, Porsolt’s Test)

The study was carried out using the test proposed by R. Porsolt [74]. The method is based on the observation of an animal forced to swim in a confined space from which there is no escape. After an initial period of vigorous attempts to get out, the animal finally gives up on escaping. The test consists of immersing the mouse individually in a cylindrical beaker (diameter 10 cm, height 25 cm) filled with water (at a temperature of 23–25 °C) to a height of 10 cm for 6 min. The immobility time, considered as the state in which the mouse performs only those movements necessary to keep its head above the water, is measured between 2 and 6 min.

#### 4.4.7. Statistical Analysis

The results were calculated by the one-way analysis of variance (ANOVA), followed by Dunnett’s post hoc test and Student’s *t*-test if needed. The results are presented as mean  ±  standard errors (SEM). The level of *p* < 0.05 was considered statistically significant. All the figures were prepared by the GraphPad Prism version 5.00 for Windows, GraphPad Software (San Diego, CA, USA; www.graphpad.com).

## 5. Conclusions

Summarizing the above results, it can be concluded that the tested compound SERAAK1 does not negatively affect the mobility of the animals and does not interfere with locomotor functions or coordination. The antidepressant-like effect demonstrated in the FST and the significant anxiolytic effect observed in the EPM test indicate a favorable pharmacological profile of the tested compound.

Overall, the integration of experimental and computational techniques provided valuable insights into the pharmacokinetics and pharmacological properties of SERAAK1. By understanding and addressing these findings in further steps of the optimization campaign, the development can proceed in a more structured, rational way, maximizing the therapeutic potential of SERAAK1 derivatives while minimizing risks.

## Figures and Tables

**Table 1:** Percentage of inhibition of the CYP3A4 activity measured in the presence of examined compounds in a concentration of 10 µM.

Examined Compound	CYP3A4 Activity Inhibition, Evaluated in the Concentration of 10 µM [% of Inhibition ± SEM]
Ketoconazole	100 ± 1
SERAAK1	−352 ± 9 ^[a]^

^[a]^ a negative value of the % of inhibition indicates a stimulating effect on the activity of the cytochrome in comparison to the base state without ligands.

**Table 2:** Percentage of inhibition of the CYP2D6 activity measured in the presence of examined compounds in a concentration of 10 µM.

Examined Compound	CYP2D6 Activity Inhibition, Evaluated in the Concentration of 10 µM [% of Inhibition ± SEM]
Quinidine	100 ± 1
SERAAK1	95 ± 1

**Table 3:** The outcomes of the PAMPA test expressed as permeability coefficient (Pe) values.

Examined Compound	P_e_ ± SD [×10^−6^ cm/s]
Caffeine	12.4 ± 0.6
Sulpiride	0.011 ± 0.002
SERAAK1	<0.010

## Data Availability

Data available on request.

## Supplementary Materials

The following supporting information can be downloaded at https://www.mdpi.com/article/10.3390/molecules30102165/s1, Figure S1: Porcupine plots obtained for protein–ligand complexes after molecular dynamics simulations. Figure S2: High-Resolution Mass Spectrometry (HRMS) Analysis of SERAAK1. Figure S3: 1H NMR Spectrum of SERAAK1 (600 MHz, MeOD). Figure S4: 13C NMR Spectrum of SERAAK1 (151 MHz, CDCl3). Figure S5: Chromatogram of SERAAK1.

## Abbreviations

The following abbreviations are used in this manuscript:

EPM	elevated plus maze test
FST	forced swim test
GPCR	G protein coupled receptor
PCA	principal component analysis
PDB	Protein Data Bank
POPC	1-palmitoyl-2-oleoyl-sn-glycero-3-phosphocholine
RMSD	root mean square deviation
RMSF	root mean square fluctuation
SPC	simple point charge
TEBA	Benzyltriethylammonium chloride
