# Supplementary material for: Synthesis, Experimental and Computational Evaluation of SERAAK1 as a 5-HT2A Receptor Ligand
Source: Molecules. 2025 May 14;30(10):2165. doi: 10.3390/molecules30102165 (PMC12114195; doi:10.3390/molecules30102165)
Supplement: Supplementary file 1 [file molecules-30-02165-s001.zip › molecules-3580540-supplementary.pdf]

Supplementary Information

Synthesis, Experimental and Computational Evaluation of SERAAK1 as a 5-HT_{2A} Receptor Ligand

Agata Zięba [1,*], Ewa Kędzierska [2], Michał K. Jastrzębski [1], Tadeusz Karcz [3], Agnieszka Olejarz-Maciej [3], Agata Sumara [4], Tuomo Laitinen [5], Tomasz M. Wróbel [1], Emilia Fornal [4], Marián Castro [6,7] and Agnieszka A. Kaczor [1,5,*]

[1] Department of Synthesis and Chemical Technology of Pharmaceutical Substances with Computer Modeling Laboratory, Faculty of Pharmacy, Medical University of Lublin, 4A Chodźki St., 20093 Lublin, Poland
[2] Department of Pharmacology and Pharmacodynamics, Faculty of Pharmacy, Medical University of Lublin, 4A Chodźki St., 20093 Lublin, Poland
[3] Department of Technology and Biotechnology of Drugs, Faculty of Pharmacy, Jagiellonian University, Medical College, Medyczna 9, 30688 Kraków, Poland
[4] Department of Bioanalytics, Medical University of Lublin, Jaczewskiego 8b St., 20-090 Lublin, Poland
[5] School of Pharmacy, University of Eastern Finland, Yliopistonranta 1, P.O. Box 1627, 70211 Kuopio, Finland
[6] Department of Pharmacology, Center for Research in Molecular Medicine and Chronic Diseases (CIMUS), Universidade de Santiago de Compostela, Avda de Barcelona, 15782 Santiago de Compostela, Spain
[7] Instituto de Investigación Sanitaria de Santiago de Compostela (IDIS), Travesía da Choupana s/n, 15706 Santiago de Compostela, Spain
* Correspondence: zieba.agata@gmail.com (A.Z.); agnieszka.kaczor@umlub.pl (A.A.K.)

Supplementary Figures

Figure S1. Porcupine plots obtained for protein – ligand complexes after molecular dynamics simulations.
Figure S2. High- Resolution Mass Spectrometry (HRMS) Analysis of SERAAK1.
Figure S3. ^{1}H NMR Spectrum of SERAAK1 (600 MHz, MeOD).
Figure S4. ^{13}C NMR Spectrum of SERAAK1 (151 MHz, $CDCl_3$).
Figure S5. Chromatogram of SERAAK1.

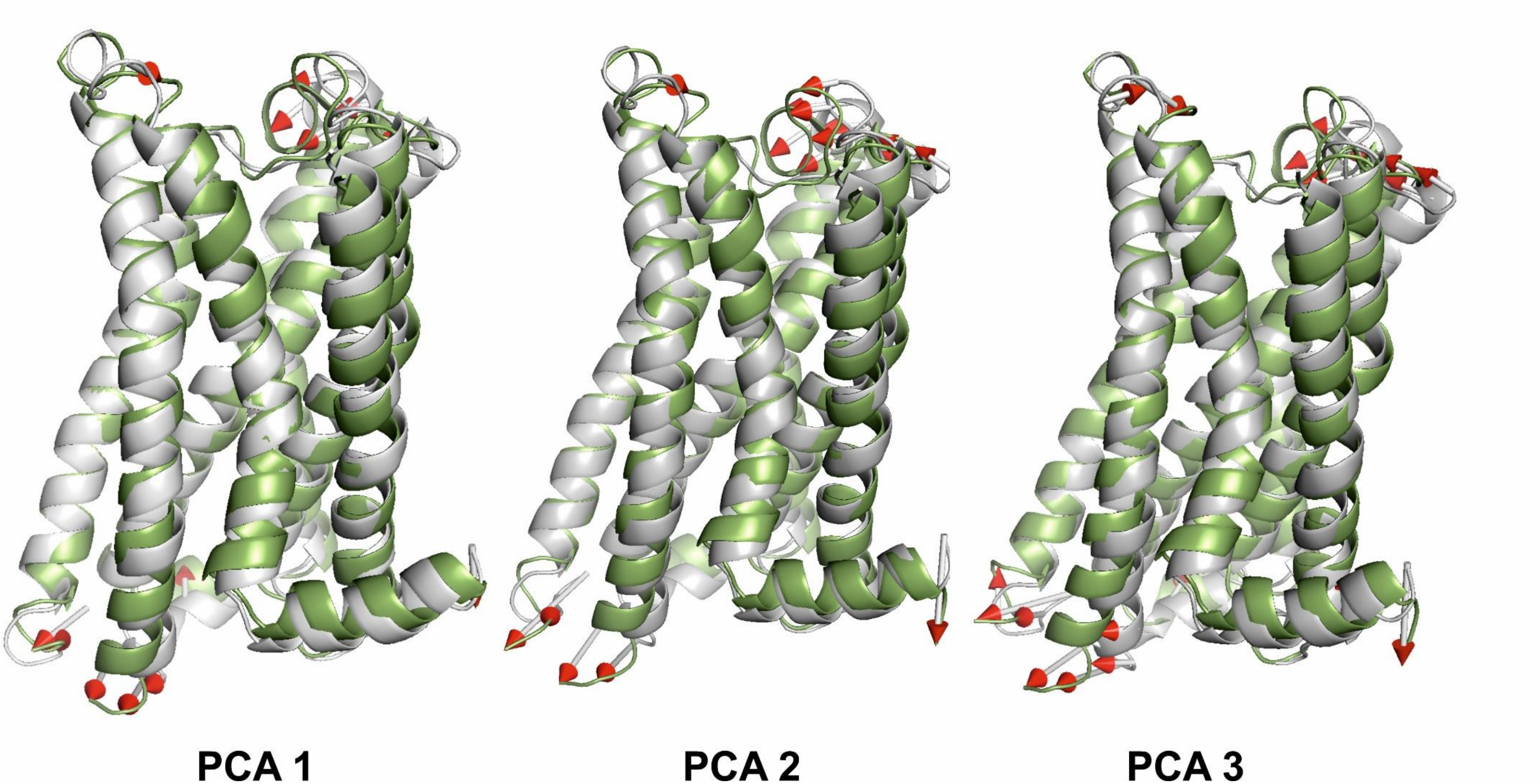

Figure S1. Porcupine plots obtained for protein – ligand complexes after molecular dynamics simulations.

**IUPAC NAME**	1-(benzhydryloxy)-3-(4-benzhydrylpiperazin-1-yl)propan—2-ol
**Chemical Formula**	C33H36N2O2
**Monoisotopic Mass:**	492.2777 Da
**[M+H]+**	493.2855 Da

Measured HRMS spectrum

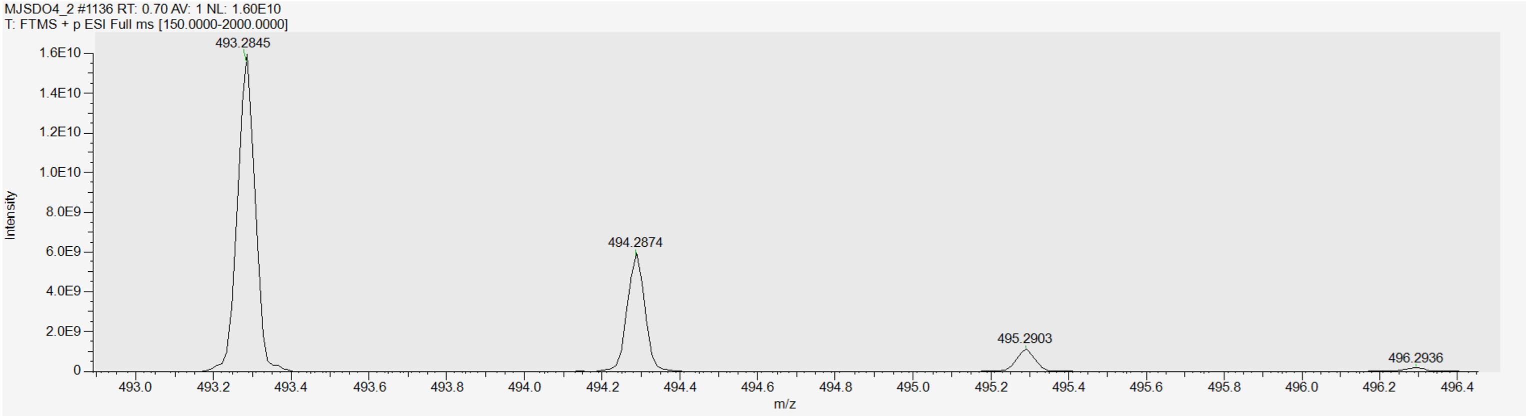

Measured Mass (Da): 493.2845

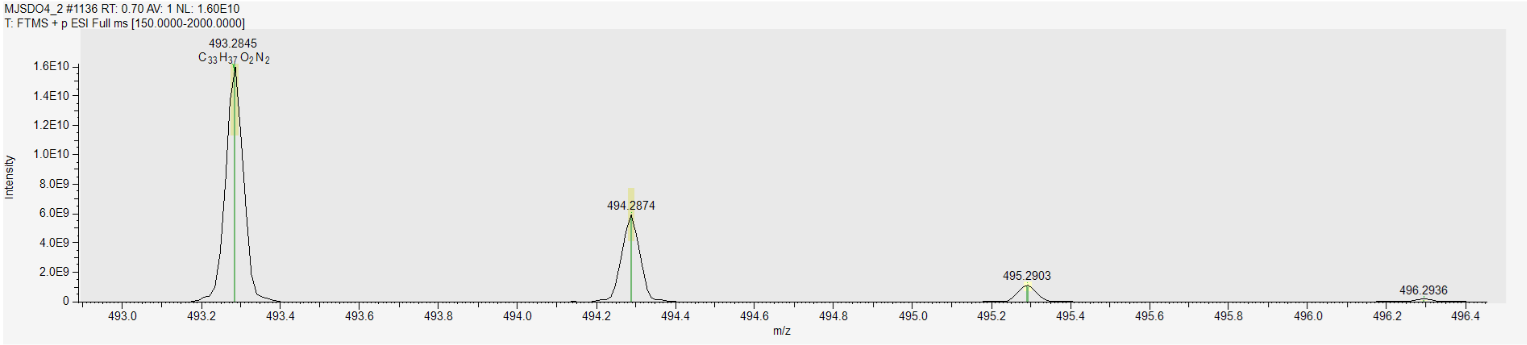

Isotopic pattern match score: 98.77%

Figure S2. High- Resolution Mass Spectrometry (HRMS) Analysis of SERAAK1.

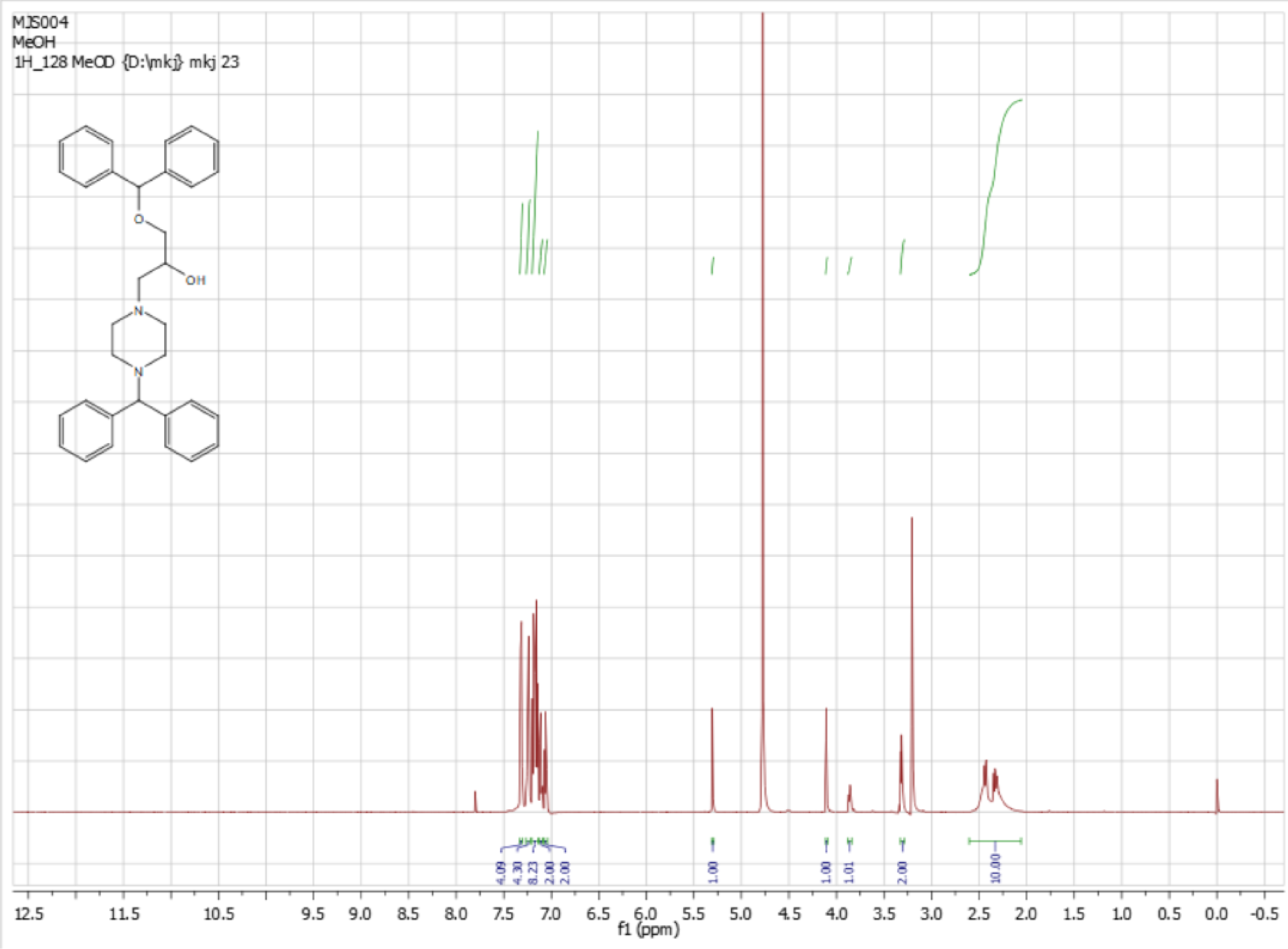

Figure S3. ^{1}H NMR Spectrum of SERAAK1 (600 MHz, MeOD).

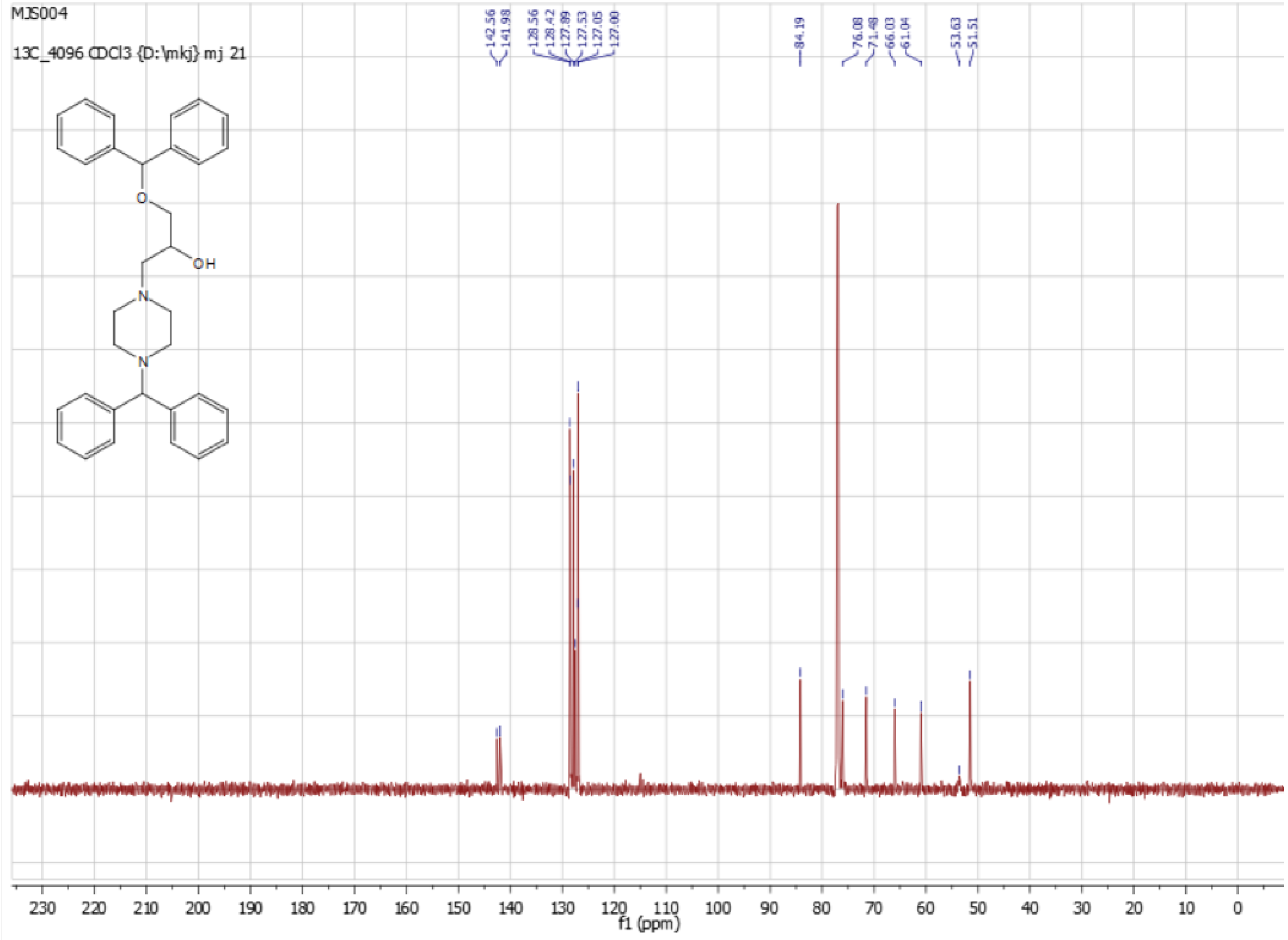

Figure S4. ^{13}C NMR Spectrum of SERAAK1 (151 MHz, $CDCl_3$).

Chromatogram and Results

Injection Details

Injection Name:	**MJS004**	*Run Time (min):*	**8.00**
Vial Number:	**GA2**	*Injection Volume:*	**6.00**
Injection Type:	**Unknown**	*Channel:*	**UV_VIS_1**
Calibration Level:		*Wavelength:*	**254**
Instrument Method:	**method7**	*Bandwidth:*	**2**
Processing Method:	**Qualitative**	*Dilution Factor:*	**1.0000**
Injection Date/Time:	**24-Apr-25 11:24**	*Sample Weight:*	**1.0000**

Chromatogram

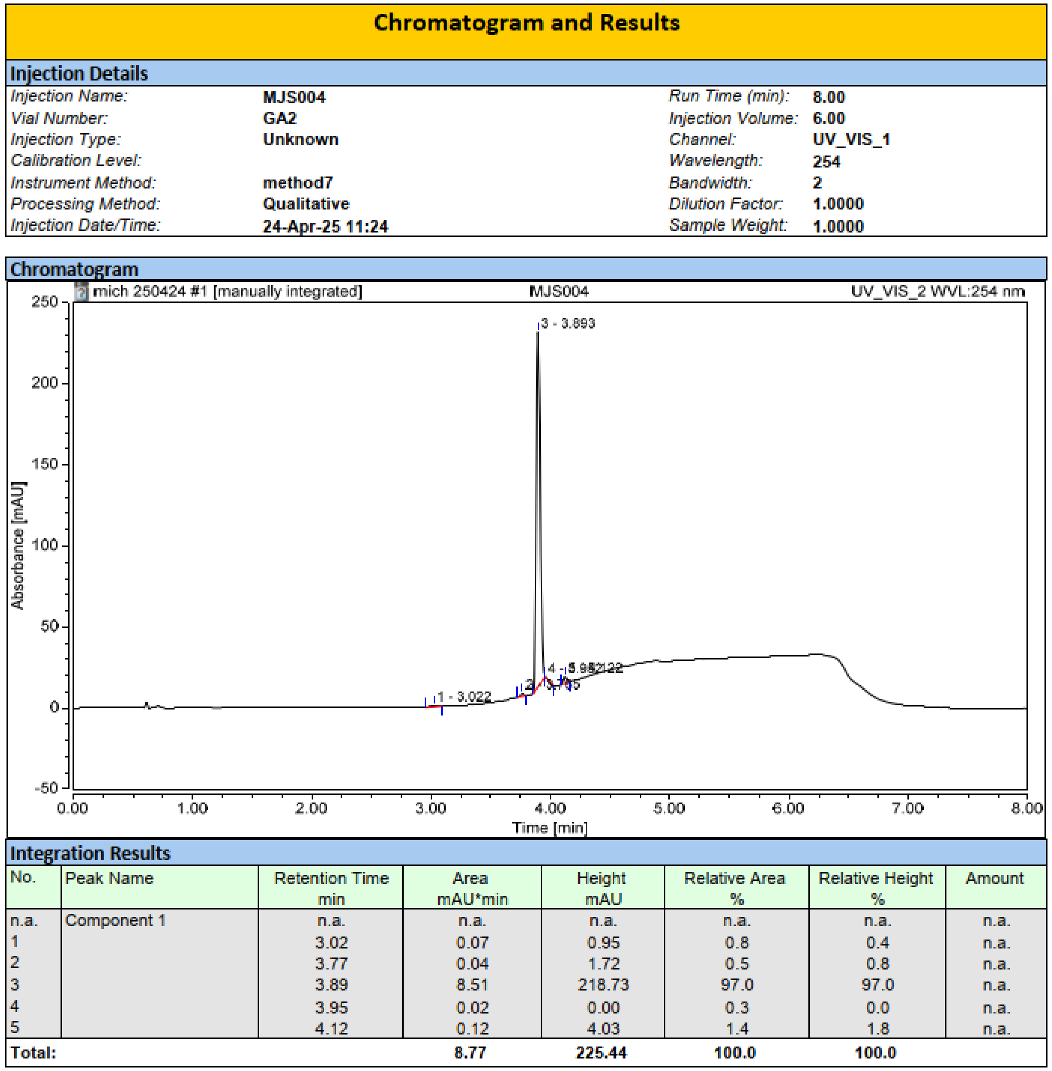

Integration Results

No.	Peak Name	Retention Time min	Area mAU*min	Height mAU	Relative Area %	Relative Height %	Amount
n.a.	Component 1	n.a.	n.a.	n.a.	n.a.	n.a.	n.a.
1		3.02	0.07	0.95	0.8	0.4	n.a.
2		3.77	0.04	1.72	0.5	0.8	n.a.
3		3.89	8.51	218.73	97.0	97.0	n.a.
4		3.95	0.02	0.00	0.3	0.0	n.a.
5		4.12	0.12	4.03	1.4	1.8	n.a.
**Total:**			**8.77**	**225.44**	**100.0**	**100.0**	

Figure S5. Chromatogram of SERAAK1.
